# Bandgap atomistic calculations on hydrogen-passivated GeSi nanocrystals

**DOI:** 10.1038/s41598-021-92936-z

**Published:** 2021-06-30

**Authors:** Ovidiu Cojocaru, Ana-Maria Lepadatu, George Alexandru Nemnes, Toma Stoica, Magdalena Lidia Ciurea

**Affiliations:** 1 National Institute of Materials Physics, 405A Atomistilor Street, 077125 Magurele, Romania; 2 Faculty of Physics, University of Bucharest, 405 Atomistilor Street, 077125 Magurele, Romania; 3 Academy of Romanian Scientists, 54 Splaiul Independentei, 050094 Bucharest, Romania

**Keywords:** Nanoparticles, Electronic structure, Quantum dots

## Abstract

We present a detailed study regarding the bandgap dependence on diameter and composition of spherical Ge-rich Ge_*x*_Si_1−*x*_ nanocrystals (NCs). For this, we conducted a series of atomistic density functional theory (DFT) calculations on H-passivated NCs of Ge-rich GeSi random alloys, with Ge atomic concentration varied from 50 to 100% and diameters ranging from 1 to 4 nm. As a result of the dominant confinement effect in the DFT computations, a composition invariance of the line shape of the bandgap diameter dependence was found for the entire computation range, the curves being shifted for different Ge concentrations by ΔE(eV) = 0.651(1 − *x*). The shape of the dependence of NCs bandgap on the diameter is well described by a power function 4.58/*d*^1.25^ for 2–4 nm diameter range, while for smaller diameters, there is a tendency to limit the bandgap to a finite value. By H-passivation of the NC surface, the effect of surface states near the band edges is excluded aiming to accurately determine the NC bandgap. The number of H atoms necessary to fully passivate the spherical Ge_*x*_Si_1−*x*_ NC surface reaches the total number atoms of the Ge + Si core for smallest NCs and still remains about 25% from total number of atoms for bigger NC diameters of 4 nm. The findings are in line with existing theoretical and experimental published data on pure Ge NCs and allow the evaluation of the GeSi NCs behavior required by desired optical sensor applications for which there is a lack of DFT simulation data in literature.

## Introduction

SiGe being one of the most studied semiconductor alloy continues to be in front of the developing researches for many microelectronics and optoelectronics applications. For high-speed SiGe CMOS technology, the SiGe heterojunction bipolar transistors achieved record performances^1,2^. On the other hand, the integrated photonics based on elements Si-Ge-Sn from group IV is experiencing a pronounced increase in the research activity in the field. Thus, the SiGe light detectors can extend their sensitivity in short-wave infrared range^3^. However, there is a low efficiency of light emission and band-edge absorption in SiGe large crystals due to the indirect bandgap character and necessary participation of phonons to the optical transitions. The efficiency can be improved by quantum confinement in NCs or by Sn alloying or by strain engineering to obtain direct bandgap in (Si)GeSn^4–8^. Versatile techniques were developed for embedding Ge and GeSi NCs in oxides for fabrication of optoelectronic devices benefiting from the advantages of materials and technology, namely the compatibility with CMOS technology and cost-effective fabrication, while being environmentally friendly. Also, they are a very advantageous alternative to III–V semiconductors for optoelectronic devices. The most interesting applications based on Ge and GeSi NCs are in optoelectronics and nanophotonics, i.e. photodetectors^9–11^, LEDs^12^, non-linear optics applications^13^ and energy harvesting devices^14,15^.

Quantum confinement effect was evidenced in small Ge and GeSi NCs^16–18^, enabling bandgap engineering along with composition^19^, shape^20^ and strain^21,22^ leading to tuning of optical and photoelectrical properties of NCs.

It was shown that two mechanisms compete in achieving no-phonon radiative transitions in NCs of Si and Ge that are indirect bandgap semiconductors in bulk. One mechanism is related to the relaxation of momentum conservation law due to the spatial confinement and Heisenberg uncertainty principle, being dominant in both Si and Ge NCs, and the other mechanism is the inter-valley coupling between direct and indirect states induced by the interface of the NC with the embedding matrix^23^. Another way of bandgap tuning is by tailoring its level of directness, as recently demonstrated direct bandgap light emission in Ge and GeSi nanowires with hexagonal structure^5,24^, and GeSi quantum dots^25^ and also by infrared detection extended to longer wavelengths in NCs of direct bandgap GeSn alloys^26^.

Strong quantum confinement effect is expected in Ge NCs as the Bohr exciton radius is 24 nm, thus facilitating the bandgap tuning by tailoring the NCs size in relatively large NCs^27,28^. Moreover, by alloying Ge with Si, GeSi NCs are more thermally stable than Ge NCs by impeding the fast diffusion of Ge during nanocrystallization by annealing^29^. Alloy GeSi NCs benefit from the complete miscibility of Ge with Si over the whole composition range, while maintaining the same crystalline structure^20^. The miscibility and intermixing of Ge and Si in GeSi random alloys can be associated to the strong self-diffusion that is theoretically explained by the vacancies formation and their contribution to the diffusion processes^30,31^. The vacancies formation energy reduces for high Ge concentration^30^ increasing the diffusion coefficient as experimentally proved^32^. By employing DFT computation it was shown that the local electronegativity of the defects is strongly dependent upon the nearest neighbor environment^33^.

Theoretical and numerical studies in the frame of DFT are valuable tools to complement experimental data and better understand the results, providing new insights in the development of nanomaterials with targeted properties.

With respect to GeSi nanostructures, it was shown based on calculated density of states (DOS) that the absorption edge of small Si and Ge NCs (0.6–1.0 nm) embedded in SiO_2_ is dependent on their size^34^. A study of structural and electronic properties of hydrogenated Si and Ge nanowires and Si, Ge, Si/Ge NCs (0.8 to 2.4 nm) shows that encapsulated Ge NCs could act as optical absorption centers in the infrared region^35^. The study on the structural stability of H-passivated GeSi NCs by calculating formation enthalpies of different Ge_x_Si_y_H_z_ isomers reveals that most stable GeSi:H NCs are the ones with the lowest formation enthalpy and widest bandgap^36^. The theoretical works from literature report results on small diameter range Ge NCs (1–2 nm), only, in contrast to experiments dealing with larger GeSi NCs^29,37–39^. Thus, there is a lack of theoretical DFT investigation on SiGe NCs of commonly experimentally observed spherical shape and for a wide range of compositions and sizes.

In this work, we present first principles electronic DOS and energy gap DFT calculations on spherical H-passivated GeSi NCs with large ranges of Ge content (50–100%) and NCs diameter (1–4 nm). A composition invariance of the line shape of the bandgap diameter dependence was found for the whole diameter and composition computation range. For comparison with experimental bandgap values and to be used for the design and characterization of optoelectronic devices based on GeSi NCs, the bandgap diameter dependences of different GeSi NC compositions are extrapolated to larger NCs by considering the asymptotic bulk values.

## Results and discussion

### Structure of spherical Ge and GeSi NCs

**Figure 1:**
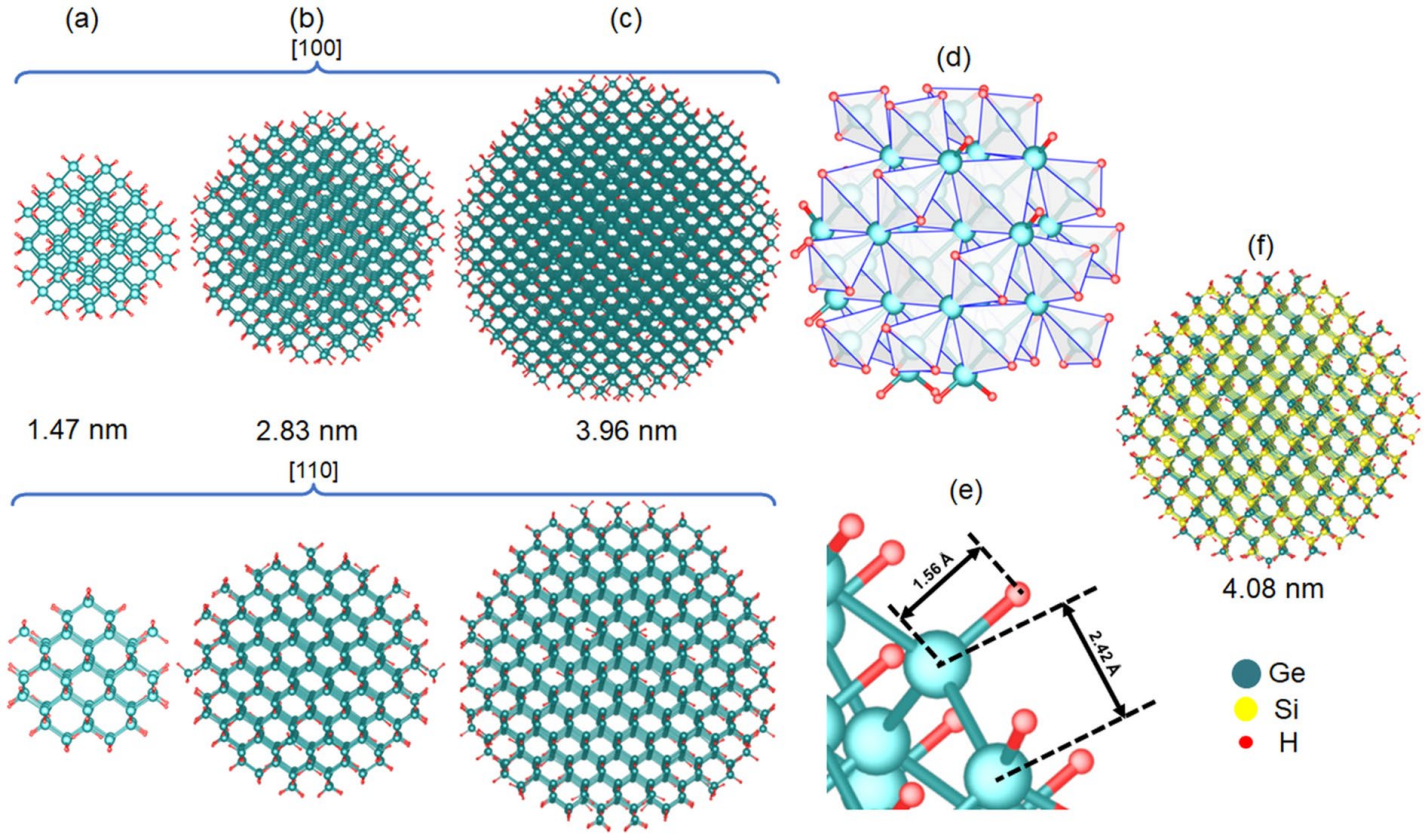
(**a**–**e**) Relaxed Ge:H NC model in atomistic simulations: (**a**) small (1.47 nm diameter), (**b**) medium (2.83 nm) and (**c**) big NCs (3.96 nm); (**d**) atomic coordinates based on Ge tetrahedral symmetry; (**e**) surface detail of relaxed Ge:H NC. (**f**) Model for relaxed GeSi:H NC with 50:50 Ge:Si composition. Models are produced by VESTA^41^.

For DFT calculations, spherical NCs of Ge and GeSi with different Ge contents (50%, 75%, 90% and 95%) were constructed using in-house software. The initial coordinates of Ge and Si atoms in NCs correspond to the bulk cubic Ge lattice (space group Fd-3m) with a lattice constant of 5.66 Å. GeSi NC is built in a similar way as Ge NC, but Ge atoms are randomly substituted by Si atoms to obtain the desired Ge concentration. The generated GeSi spheres have diameters in the 1.25–3.96 nm range. For the *x* = 100% Ge case, Fig. 1a﻿﻿–c shows small, medium and large Ge NCs, respectively, projected along [100] and [110] directions. Facets are clearly revealed especially for images of the [100] orientation.

The surface of both Ge and GeSi NCs was passivated with H atoms (annihilating dangling bonds effects in Ge:H and GeSi:H NCs). In the Ge NC, the H atoms are positioned in the vertices of Ge tetrahedron or out of tetrahedron depending on the number of Ge dangling bonds (Fig. 1d). Similarly, in GeSi NC, H atoms are positioned at Ge or Si dangling bonds. After geometrical construction, the H-passivated NCs are subjected to the energetic relaxation by atomistic computations as described in the “Methods” section. In a relaxed Ge NC, the Ge–H bond length is 1.56 Å, while Ge–Ge length is 2.42 Å close to the bulk value of 2.45 Å^40^, as seen in the surface detail of Fig. 1e.

The geometric construction of energetically relaxed H-passivated GeSi NC with *x* = 50% is exemplified in Fig. 1f. Further details are given in “Methods” section and Supporting Information (SI)—Table S1.

**Figure 2:**
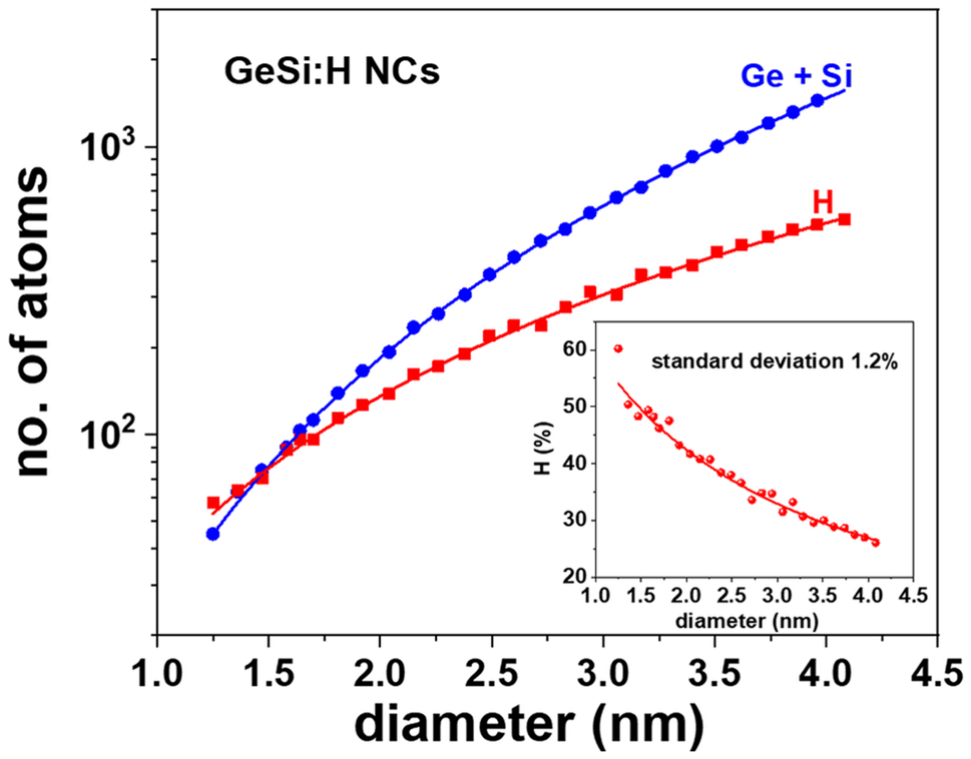
NC diameter dependences of the number of Ge and substituting Si atoms (blue dots), and the number of H atoms (red squares) necessary to construct the model of GeSi:H NCs with fully passivated surface; the continuous lines correspond to 44.0 atoms/nm^3^ atomic density for Ge and Si and to 10.8 atoms/nm^2^ for H surface density uniformly distributed on spherical GeSi:H NCs. The inset shows the concentration of H passivation atoms in NC.

The passivation of NC surface is mandatory for excluding any surface localized states near the band edges as the calculations focus on the bandgap^23^, but also to ensure the computation convergence. The diameter dependences of the number of H atoms and the number of Ge and Si atoms in each NC (listed in Table S1 in SI) resulting from the model are shown in Fig. 2. In Fig. 1, one can see that Ge and Si atoms in NCs do not perfectly fill the spherical shape. However, the mean density of 44.0 atoms/nm^3^ of Ge and substituting Si atoms (Fig. 2) uniformly distributed in spherical NCs that fits well the diameter dependence of the constructed NCs is slightly smaller than Ge bulk value of 44.1 atoms/nm^3^.

It is obvious that these results are independent of Si at.% content taking into account the similar way in which the atomic coordinates of both Ge and GeSi NCs are generated. The concentration of H at.% required for complete passivation of the NC surface reaches quite high values, more than 50% for small diameters and is still 25% high for the NC size of 4 nm and corresponds to the mean value of 10.8 atoms/nm^2^ in the spherical NC (inset in Fig. 2).

### Diameter dependence of the Ge NCs bandgap

**Figure 3:**
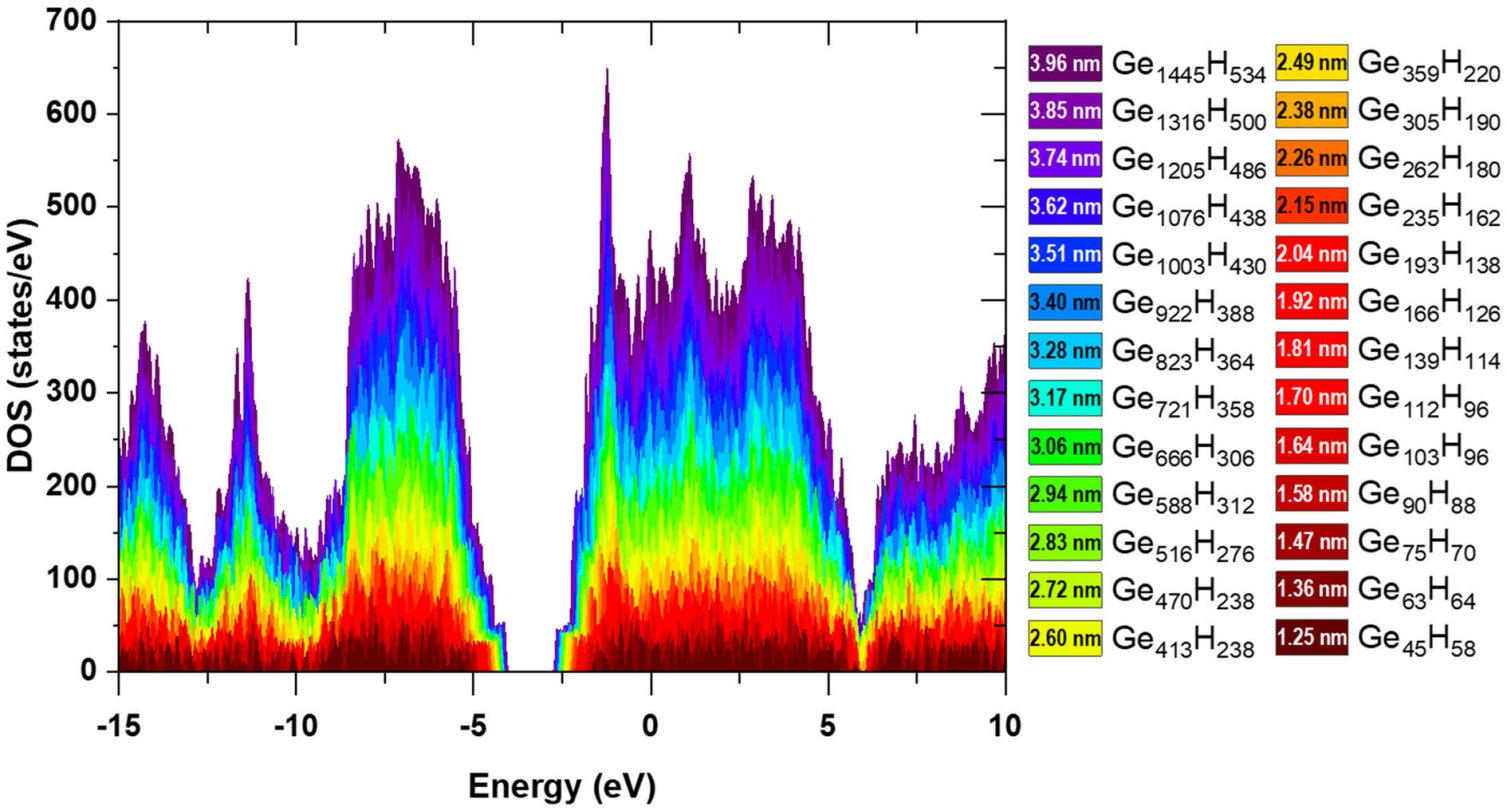
Cumulative DOS of Ge NCs with diameters in the 1.25–3.96 nm range (indices denote the number of Ge and H atoms).

Spherical Ge NCs passivated with H atoms, with diameters from 1.25 to 3.96 nm were considered in the atomistic simulation. A cumulative DOS plot of Ge NCs is shown in Fig. 3, revealing the decrease of energy gap with the increase of NC diameter.

**Figure 4:**
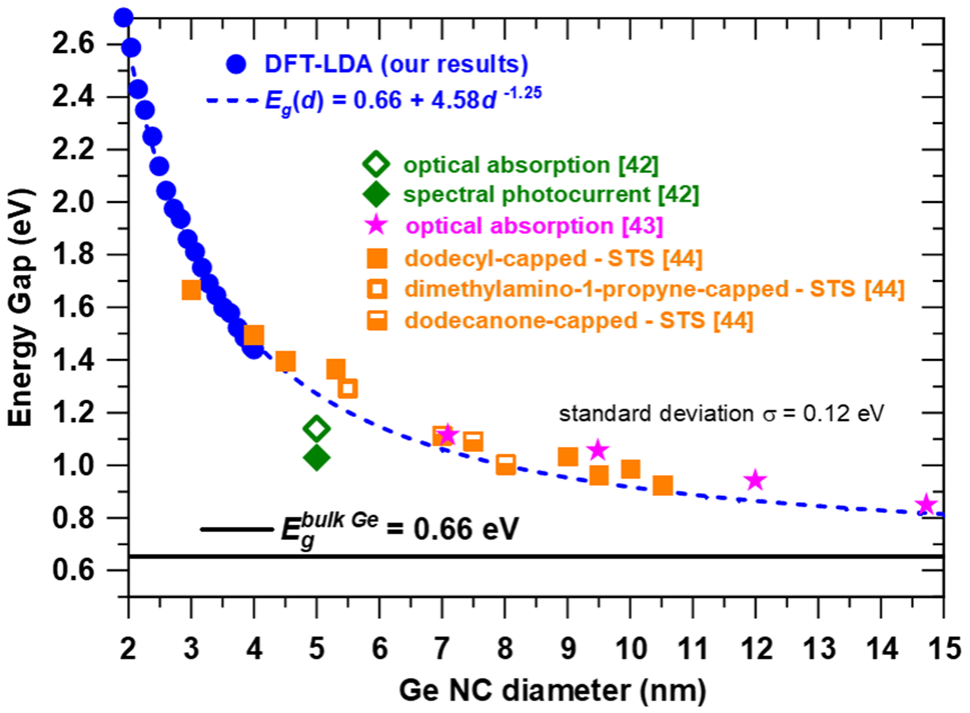
Ge NCs energy gap dependence on diameter: DFT calculations (filled blue circles for calculated points and dashed blue line for fit function in Eq. 1) and experimental data from optical absorption^42,43^, spectral photocurrent^42^ and scanning tunneling spectroscopy (STS)^44^ for which filled, empty and half-filled squares correspond to different capping ligands.

The calculated energy gap *E*_*g*_ of Ge NCs is presented in Fig. 4 and in Table 1. The energy gap dependence on diameter can be described by a power law asymptotic to the bandgap of bulk Ge, for diameters higher than 2 nm:

1 $$E_{g} \left( d \right) = E_{g}^{{bulk}} + Ad^{{ - \alpha }}$$

where *α* = 1.25 ± 0.02 and *A* = 4.58 ± 0.14 eV are the fit parameters, *d* is expressed in nm and *E*_*g*_^*bulk*^ = 0.66 eV is the experimental value, close to theoretical bandgap of 0.63 eV calculated by us using DFT in local density approximation (LDA). The accuracy assessment of LDA and generalized gradient approximation (GGA) methods is made by band structure computation of bulk Ge and Si. As can be seen in Figure S1 in SI the LDA computation gives a smaller bandgap for Ge with a low error of 30 meV in respect to the experimental value, while the GGA gives a much lower value of bandgap of 0.47 eV (200 meV error). For bulk Si, both methods give a bandgap almost 200 meV higher than the experimental value. Therefore, for the DOS calculation in GeSi NCs presented in the next section, we used the LDA calculation method and we limited the composition to the range of 50—100% Ge.

**Table 1:** Calculated energy gap *E*_*g*_ for H-passivated Ge_*x*_Si_1−*x*_ NCs.

*d* (nm)	*E*_*g*_ (eV)				
	Ge *x* = 100%	95%	90%	75%	50%
1.25	3.68	3.72	3.85	3.87	4.04
1.36	3.58	3.60	3.61	3.70	3.91
1.47	3.44	3.44	3.49	3.57	3.73
1.58	3.26	3.28	3.29	3.38	3.50
1.70	3.04	3.05	3.11	3.21	3.35
1.81	2.81	2.83	2.85	2.95	3.12
1.92	2.70	2.75	2.77	2.85	3.01
2.04	2.59	2.61	2.65	2.70	2.88
2.15	2.43	2.47	2.50	2.61	2.77
2.26	2.35	2.40	2.42	2.51	2.66
2.38	2.25	2.29	2.31	2.43	2.58
2.49	2.14	2.19	2.21	2.31	2.47
2.60	2.04	2.09	2.12	2.22	2.38
2.72	1.98	2.03	2.08	2.13	2.33
2.83	1.94	1.96	1.99	2.11	2.27
2.94	1.86	1.89	1.93	2.02	2.21
3.06	1.81	1.84	1.87	1.97	2.16
3.17	1.75	1.79	1.81	1.89	2.10
3.28	1.69	1.72	1.76	1.85	2.02
3.40	1.65	1.68	1.71	1.80	1.96
3.51	1.60	1.63	1.66	1.77	1.93
3.62	1.58	1.60	1.64	1.72	1.90
3.74	1.52	1.55	1.59	1.68	1.87
3.85	1.48	1.51	1.55	1.63	1.85
3.96	1.45	1.49	1.52	1.60	1.80

If the 0.63 eV theoretical gap is considered, the fit results are quite similar to that of the asymptotic experimental value of 0.66 eV. The fit curve extrapolated to higher diameters (15 nm) is shown in Fig. 4. One can see that our results fit well the experimental values from literature obtained by different methods such as optical absorption^42,43^, spectral photocurrent^42^ and scanning tunneling spectroscopy (STS)^44^. The standard deviation of the experimental results with respect to the fit has a reasonable value of σ = 0.12 eV, taking into account that the experimental NCs usually have a quite broad size distribution.

**Figure 5:**
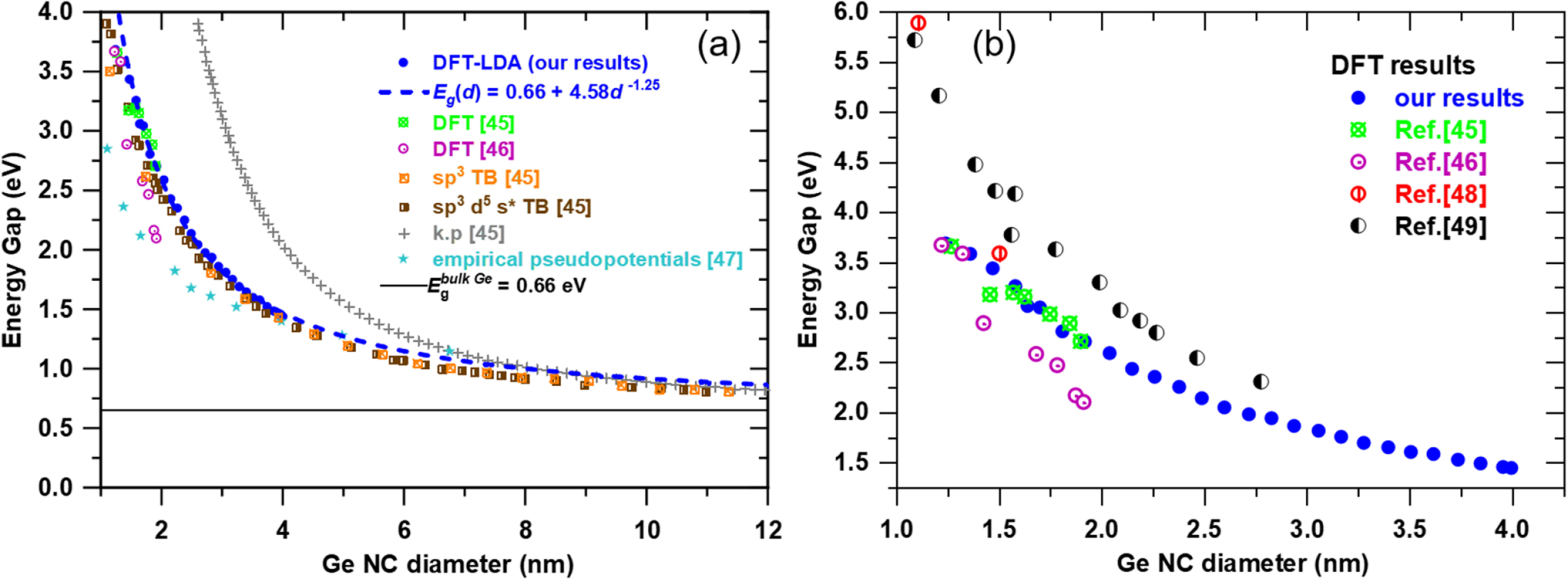
Energy gap dependence on diameter obtained in the frame of different calculations: (**a**) our DFT results (blue filled circles for calculated points and dashed line for the power law dependence in Eq. 1) compared to reported results obtained by DFT^45,46^, TB^45^,$\mathbf{k}\cdot \mathbf{p}$^45^ and empirical pseudopotentials^47^; (**b**) our DFT data compared to others^45,46,48,49^.

Figure 5a shows a comparison of our calculated energy gaps (DFT–LDA) with other theoretical energy gaps for Ge NCs from literature obtained by using DFT^45,46^, tight binding (TB)^45^, $\mathbf{k}\cdot \mathbf{p}$ and empirical pseudopotentials methods^47^. One can remark that the power law dependence (Eq. 1) overlaps the whole 1–12 nm diameter interval with the TB results^45^. Other computation methods show significant deviation from our results at small diameters (e.g. empirical pseudopotentials and $\mathbf{k}\cdot \mathbf{p}$), but all methods show a good agreement at diameters higher than ~ 8 nm.

The comparison of DFT results for the 1–4 nm diameter range is shown in Fig. 5b. One can see that we obtain similar results with those from Ref^45^ that were however obtained only for very small diameters in the 1.27–1.90 nm range.

Related to the effect of passivation on the bandgap, it was experimentally shown^44^ that the effect of capping ligand is not measurable, but it can change the Fermi level within the band gap. Our computations have shown that the Fermi level of H-passivated Ge NCs is close to the middle of the bandgap, as expected for intrinsic and well passivated semiconductor NCs. In fact, the Fermi level can be significantly moved towards conduction or valence bands by doping defects (vacancies or dopant atoms).

The type of passivation is of a great importance for the optoelectronic properties of the NCs. In experiments, NCs are often embedded in a dielectric matrix, the surface not being H-passivated necessarily. By H-passivation of NC surface, the effect of surface states near the band edges is excluded aiming to accurately determine NC bandgap. The H-passivation of spherical NCs is easily performed using our in-house developed code for the construction of NCs and has the advantage to fully passivate the free Ge bonds at NCs surface, as well as to remove the surface states from the bandgap region that is similar to the effect that takes place in amorphous hydrogenated Si. The software can also be used for O-passivation by replacing H atoms, but in this case the O atoms are placed only at sides where two Ge free bonds can be passivated by a single O atom, the rest of free Ge bonds on the NC surface remaining passivated by H atoms. We expect O–Ge bonds to produce stronger deformation of the Ge lattice at the NCs surface, resulting in some changes of the bandgap in respect to the case of the passivation with H only. DFT computations in progress will show the effect of the type of passivation atoms on the NCs bandgap.

### Diameter dependence of the GeSi NCs bandgap

Besides NC size, the alloy Ge_*x*_Si_1−*x*_ NCs provide an additional parameter, namely the composition for bandgap control over a spectral range wider than that of Ge NCs. We focus our attention on Ge-rich Ge_*x*_Si_1−*x*_ NCs because of the interest to keep the optical absorption edge and bandgap related cut-off wavelength of spectral photocurrent at longer wavelengths to be compared with reported experimental results up to 1700 nm wavelengths^29^. Thus, in DFT calculations of DOS and energy gap, we consider Ge at.% concentrations of 95, 90 and 75 at.% Ge in H-passivated GeSi NCs. The results are compared with those for stoichiometric GeSi (50:50) and pure Ge NCs by varying the NC diameter *d* from 1.25 to 3.96 nm as presented in Table 1. Due to the constraint of the numerical construction of spherical Ge_*x*_Si_1−*x*_ NCs, the Ge concentration cannot be kept strictly constant for the whole range of diameters, the standard deviation being about 0.3% (Table S1 in SI).

**Figure 6:**
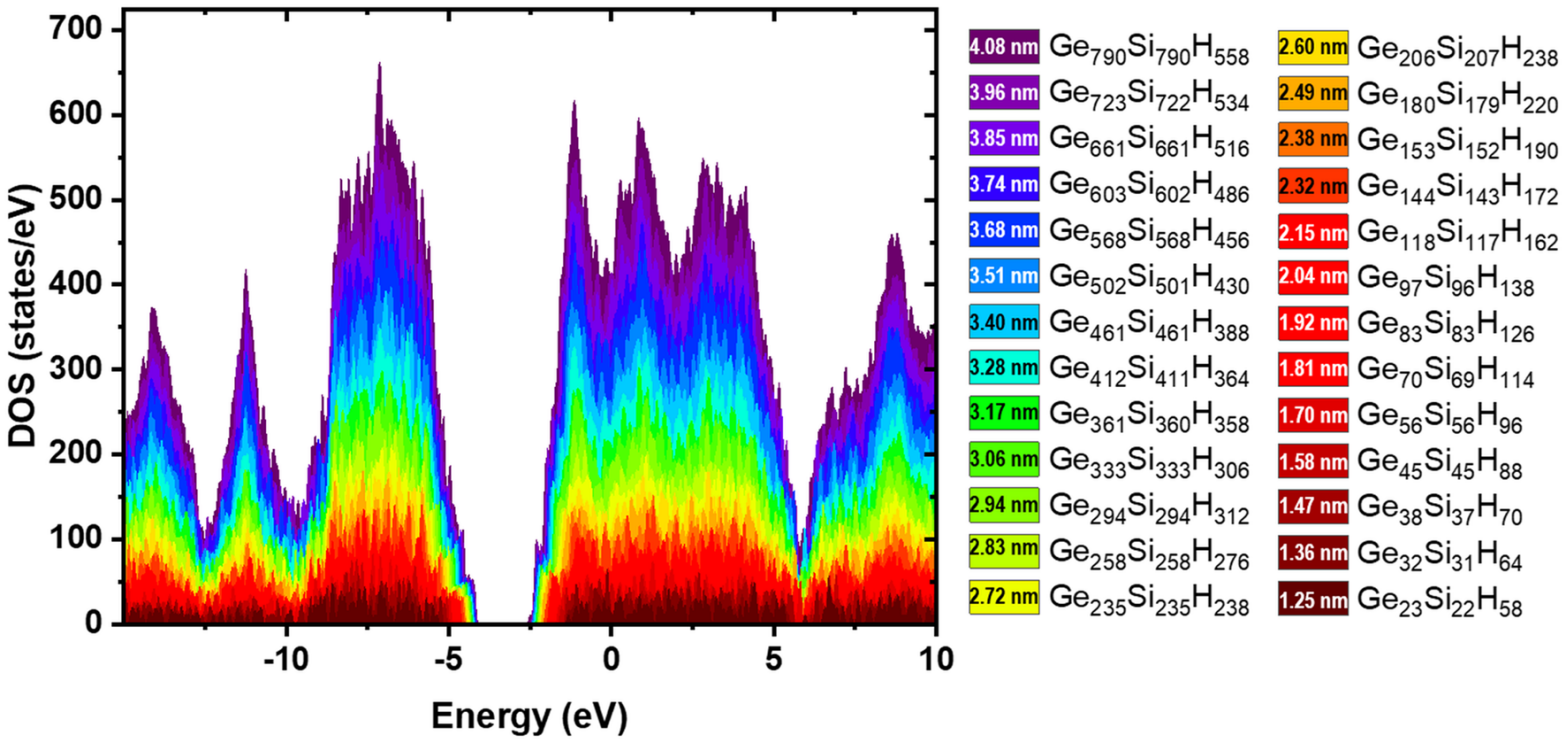
DOS for H-passivated spherical Ge_0.5_Si_0.5_ NCs.

Cumulative calculated DOS spectra for H-passivated GeSi NCs with 50% Ge concentration are given in Fig. 6 showing the energy gap decrease with diameter increase similarly as for Ge NCs.

**Figure 7:**
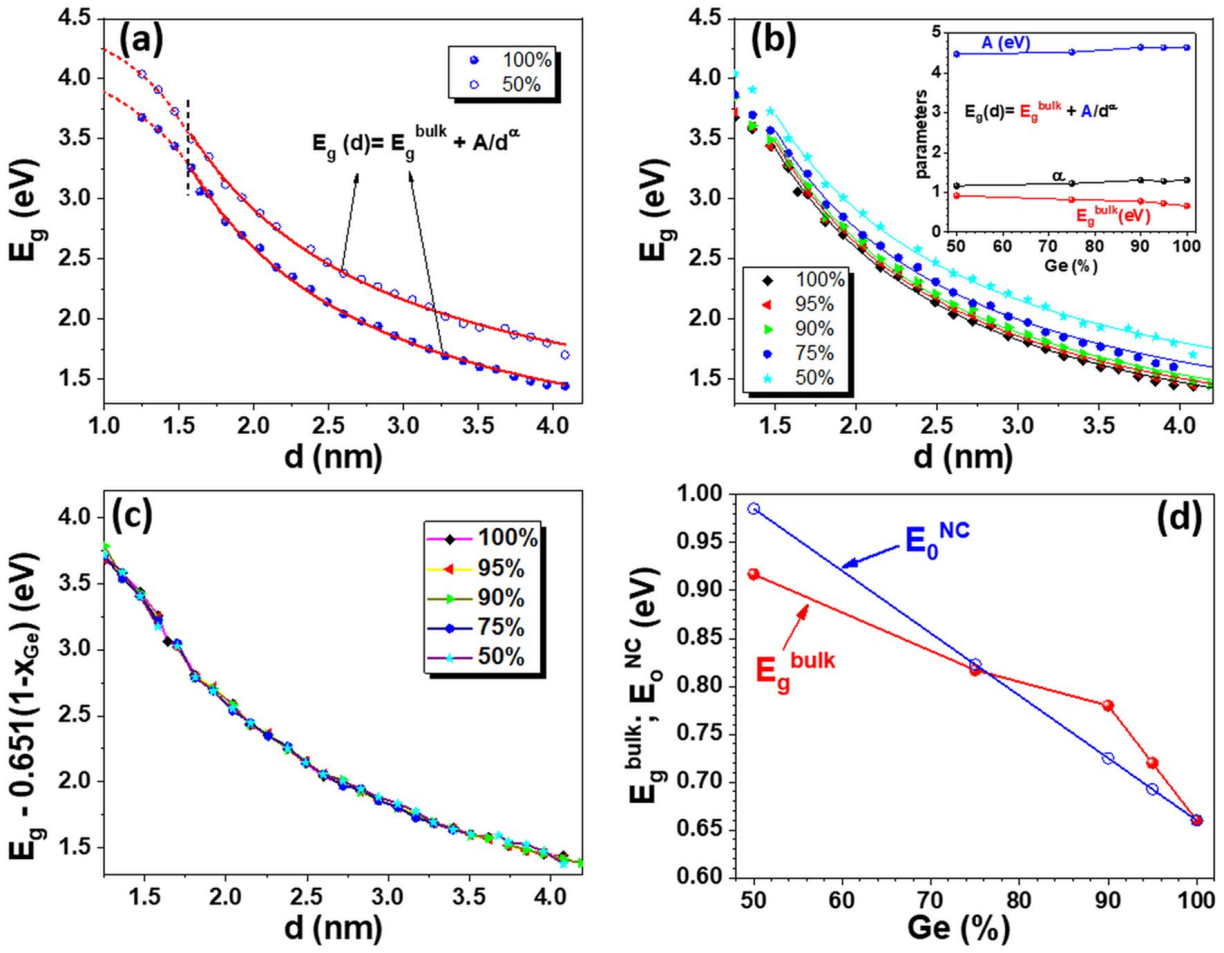
(**a**) and (**b**) Diameter dependence of bandgap *E*_*g*_(*d*) of Ge_*x*_Si_1−*x*_ NCs for different Ge at.% concentrations obtained by DFT calculations: symbols—calculated points, fit curve—continuous lines with Eq. (1); fit parameters *A* and *α*—inset in figure (**b**); *E*_*g*_ fit curves show a tendency of upper limitation for diameters smaller than 2 nm—dashed lines in figure (**a**). (**c**) Size dependences of the computed *E*_*g*_(*d*) shifted on vertical by subtracting 0.651(1 − *x*) in eV units: *E*_*g*_(*d*,*x*) = *E*_*0*_^NC^(*x*) + *A*/*d*^*α*^, *E*_*0*_^NC^ = 0.66 + 0.651(1 − *x*), *A* = 4.58 eV, *α* = 1.25; (**d**) *E*_*0*_^NC^ parameter of the cumulative fit of all computed data is compared with *E*_*g*_^*bulk*^ of Ge_*x*_Si_1−*x*_ alloys from Ioffe data^50^.

The calculated diameter dependence of the bandgap of GeSi (50:50) NC is compared to that for Ge NCs in Fig. 7a. In a similar way as for Ge NCs, the computed energy gap values for each diameter are well described by the empirical power law dependence (Eq. 1) for diameters higher than 2 nm. As can be seen (Fig. 7a), for both cases at diameters smaller than 2 nm, the increase of the bandgap shows a tendency of upper limitation that can be caused by the finite value of the difference between bonding and antibonding energies.

The fit curves using Eq. (1) for each Ge concentration of GeSi NC are shown in Fig. 7b, and corresponding fit parameters *A*, *α* and *E*_*g*_^*bulk*^ are given in inset. The experimental dependences of *E*_*g*_^*bulk*^ (dependent on Ge concentration) for asymptotic values are taken from the Ioffe database^50^ (also in Fig. 7d). Moderate fluctuations of the fit parameters *A* and *α* are obtained (inset in Fig. 7b). This suggests that the shape of diameter dependence curve of GeSi NCs energy gap is slightly dependent on the Ge concentration over the explored composition range. Indeed, if all computed *E*_*g*_(*d*) curves are vertically shifted by subtracting 0.651(1 − *x*) term dependent on Ge concentration *x*, the data are well superposed over the curve for Ge NCs, shown in Fig. 7c. It results that the computed band gap *E*_*g*_(*d,x*) of Ge_*x*_Si_1−*x*_ NCs as a function of diameter *d* and Ge concentration *x* can be approximated by *E*_*g*_(*d,x*) = *E*_*g*_^Ge^(*d*) + 0.651(1 − *x*) in which *E*_*g*_^Ge^(*d*) is the bandgap diameter dependence for pure Ge NCs. This invariance of the shape of the computed bandgap valid for the whole explored diameters (1.6–3.9 nm) and composition range (Fig. 7c) is well described by a power function as that of Eq. (1):

2 $$E_{g} \left( {d,x} \right) = E_{0} ^{{{\text{NC}}}} + {\text{4}}.{\text{58}}/d^{{{\text{1}}.{\text{25}}}}$$

with *E*_0_^NC^ = 0.66 + 0.651(1 − *x*) instead of *E*_*g*_^*bulk*^ (Eq. 1). This asymptotic *E*_0_^NC^ to larger NCs deviates from the bulk value *E*_*g*_^*bulk*^ up to 0.1 eV and does not reflect the influence of the change from L to X minimum of the conduction band in bulk GeSi alloys (the knee observed at 90% Ge in Fig. 7d). It is what we can expect as a result of confinement effect in NCs that removes the indirect bandgap character by relaxation of the momentum conservation law.

## Conclusions

Spherical H-passivated Ge-rich Ge_*x*_Si_1−*x*_ NCs with Ge atomic concentration in the range of 50–100% and diameters from 1 to 4 nm were theoretically studied by atomistic DFT calculations using the SIESTA software. The results serve to fill the gap in literature regarding theoretical investigations on electronic band structure of GeSi NCs. The GeSi NCs were constructed using an in-house developed code by taking into account the tetrahedral symmetry (space group Fd-3m) of cubic Ge(Si). The spheres have been filled with Ge and Si atoms in the cubic Ge coordination and subjected to lattice relaxation after the H-passivation of the NCs surface. The number of H atoms necessary to fully passivate the NC surface reaches the number of Ge core atoms for smallest NCs and still remains of about 25% for 4 nm size. The computed DOS for different concentrations and diameters has been used for obtaining the NC bandgap as a function of the diameter for different Ge concentrations. It was found that the shape of *E*_*g*_(*d,x*) is almost independent on composition for the whole investigated diameter-composition range being described by *E*_*g*_(*d,x*) = *E*_*g*_^Ge^(*d*) + 0.651(1 − *x*), in which *E*_*g*_^Ge^(*d*) is the bandgap of Ge:H NCs. For diameters from 1.6 to 4 nm, the shape of the bandgap dependence on diameter can be fitted by a power function *E*_*g*_^Ge^(*d*) = 0.66 + 4.58/*d*^1.25^, and shows a tendency of upper bandgap limitation for smaller diameters. The extrapolation to the bulk bandgap value of *E*_*g*_(*d,x*) fit curves can be used for the design and characterization of optoelectronic devices based on GeSi NCs.

## Methods

All atomistic simulations were performed using the SIESTA code^51^ in order to take advantage of the localized basis functions (numerical atomic orbitals) leading to linear scaling of computation time with the dimensions of the atomistic model. The computations were performed with LDA for the exchange–correlation energy functional.

The atomic coordinates of spherical Ge_*x*_Si_1−*x*_ NC (for each considered Ge concentration *x*) were generated by considering a cubic simulation cell of 10 nm edge length in which the NC surrounded by vacuum is centered. The simulation cell acts as the unit cell in periodic boundary conditions computations. This configuration is necessary because the overlap of electronic wave functions belonging to neighboring NCs must vanish.

LDA pseudopotentials for valence electrons 4*s*^2^ 4*p*^2^ of Ge, 3*s*^2^ 3*p*^2^—Si and 1*s*^1^—H were employed, respectively. The calculations were done using a double-zeta basis size, i.e. two basis functions for each numerical atomic orbital, with a 150 Ry mesh cut-off, a limit of 50 steps for self-consistent field loop with a convergence tolerance of 10^–3^ for the elements of the density matrix and 10^–4^ for the elements of the Hamiltonian matrix. The convergence criteria refer to the simultaneous self-consistency of the density matrix and the Hamiltonian matrix. The self-consistency of the density/Hamiltonian matrix is achieved when the maximum difference between the output and the input on each element of the density/Hamiltonian matrix in a SCF loop is smaller than the respective tolerance. The passivation of NC surface is mandatory for excluding surface localized states, but also to ensure the convergence of the DFT-SIESTA computation. According to folding zone theory, for NCs the calculations are done in Γ point, only.

Total energy was computed by diagonalization of the effective Kohn–Sham Hamiltonian, for 300 K electronic temperature of the occupation function (Fermi–Dirac distribution). For geometrical relaxation of NCs, we employed the conjugate gradient method with a maximum atomic displacement of 0.1 Å and the stop criteria of either 50 relaxation steps or a force tolerance of 0.1 eV/Å. DOS was calculated for each NC, per unit energy and per unit volume, ignoring electronic spin, as $g\left(E\right)=\sum _{i}\delta \left({\varepsilon }_{i}-E\right)$, where ${\varepsilon }_{i}$ are the eigenvalues of the effective Kohn–Sham Hamiltonian, in the range between − 15 eV and 10 eV using 1000 points, with a broadening of 0.150 meV. The set of eigenvalues yields the energy gap as the difference of the two eigenvalues adjacent to the Fermi level.

## Supplementary Information

**Supplementary Information**.
