# Supplementary material for: Bandgap atomistic calculations on hydrogen-passivated GeSi nanocrystals
Source: Sci Rep. 2021 Jun 30;11:13582. doi: 10.1038/s41598-021-92936-z (PMC8245600; doi:10.1038/s41598-021-92936-z)
Supplement: Supplementary file 1 — Supplementary Information. [file 41598_2021_92936_MOESM1_ESM.pdf]

Bandgap atomistic calculations on hydrogen-passivated GeSi nanocrystals

Supporting Information

Ovidiu Cojocaru[1,2], *Ana-Maria Lepadatu*[1], *George Alexandru Nemnes*[2],

Toma Stoica[1, *], *Magdalena Lidia Ciurea*[1,3,*]

[1] National Institute of Materials Physics, 405A Atomistilor Street, 077125 Magurele, Romania

[2] University of Bucharest, Faculty of Physics, 405 Atomistilor Street, 077125 Magurele, Romania

[3] Academy of Romanian Scientists, 54 Splaiul Independentei, 050094 Bucharest, Romania

[*] Corresponding authors: Dr. T. Stoica – toma.stoica@infim.ro and Dr. M.L. Ciurea – ciurea@infim.ro

Band structure for bulk Ge and Si by DFT-LDA calculations

As a reference, we calculated band structure and bandgap of bulk Ge and Si in the frame of DFT. The indirect bandgap energy of bulk cubic Ge was calculated in both LDA and generalized gradient approximation (GGA) as shown in **Figure S1a, b**. One can see that LDA gives an accurate result, i.e. E_g^{bulk} = 0.63 eV in good agreement with the well-known value of 0.66 eV for 300 K (**Figure S1a)**. Instead, the electronic band structure computed by GGA-DFT Ge shows the bandgap with about 200 meV lower value (**Figure S1b)**. For bulk Si, both methods give almost 200 meV higher bandgap than the experimental value (**Figure S1c,d)**.

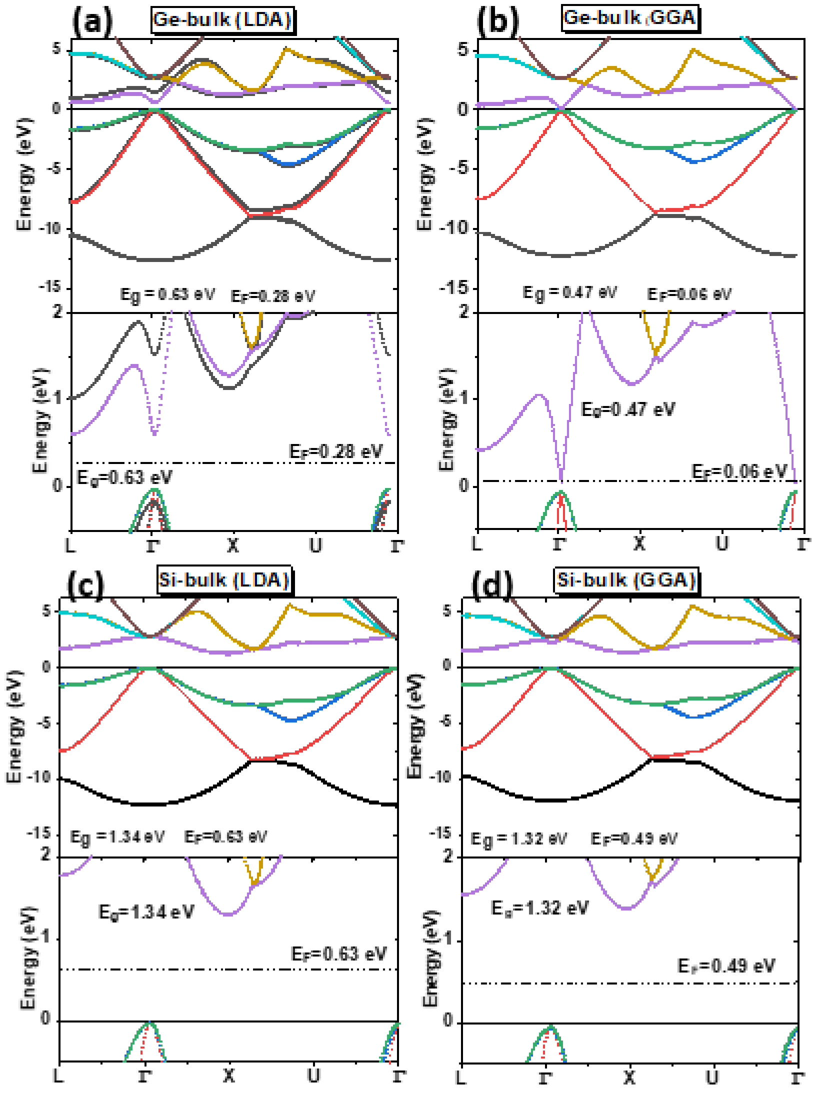

Figure S1. Band structure of bulk cubic Ge (a,b) and Si (c,d) obtained by using LDA and GGA (bottom parts of the graphs represent a zoom of the band structure in the bandgap region).

Effective Si concentration of spherical H-pasivated GeSi NCs

The geometric construction of Ge_xSi_{1-x} spherical NCs consists in filling of a sphere with Ge(Si) atoms in a lattice (space group Fd-3m symmetry). Due to the constrain of the spherical shape and of the finite number of the constituent atoms, there is a necessary small deviation from the desired composition Si at%, depending on the NC diameter, as shown in **Table S1**.

Table S1. Geometric construction of GeSi:H NCs with diameter *d*: number of constituent atoms for each atomic species and concentration together with effective Si atomic percent.

***d* (nm)**	**No of H atoms**	**No. of Ge atoms**					**No. of Si atoms**				**Effective Si concentration (at.%)**			
		**Si**: 0%	5%	10%	25%	50%	**Si**: 5%	10%	25%	50%	5%	10%	25%	50%
**1.25**	58	45	43	41	34	23	2	4	11	22	4.4	8.9	24.4	48.9
**1.36**	64	63	60	57	48	32	3	6	15	31	4.8	9.5	23.8	49.2
**1.47**	70	75	72	68	57	38	3	7	18	37	4.0	9.3	24.0	49.3
**1.58**	88	90	86	81	68	45	4	9	22	45	4.4	10.0	24.4	50.0
**1.70**	96	112	107	101	84	56	5	11	28	56	4.5	9.8	25.0	50.0
**1.81**	114	139	133	126	105	70	6	13	34	69	4.3	9.4	24.5	49.6
**1.92**	126	166	158	150	125	83	8	16	41	83	4.8	9.6	24.7	50.0
**2.04**	138	193	184	174	145	97	9	19	48	96	4.7	9.8	24.9	49.7
**2.15**	162	235	224	212	177	118	11	23	58	117	4.7	9.8	24.7	49.8
**2.26**	172	262	249	236	197	144	13	26	65	143	5.0	9.9	24.8	49.8
**2.38**	190	305	290	275	229	153	15	30	76	152	4.9	9.8	24.9	49.8
**2.49**	220	359	342	324	270	180	17	35	89	179	4.7	9.7	24.8	49.9
**2.60**	238	413	393	372	310	207	20	41	103	206	4.8	9.9	24.9	49.9
**2.72**	238	470	447	423	353	235	23	47	117	235	4.9	10.0	24.9	50.0
**2.83**	276	516	491	465	387	258	25	51	129	258	4.8	9.9	25.0	50.0
**2.94**	312	588	559	530	441	294	29	58	147	294	4.9	9.9	25.0	50.0
**3.06**	306	666	633	600	500	333	33	66	166	333	5.0	9.9	24.9	50.0
**3.17**	358	721	685	649	541	361	36	72	180	360	5.0	10.0	25.0	49.9
**3.28**	364	823	782	741	618	412	41	82	205	411	5.0	10.0	24.9	49.9
**3.40**	388	922	876	830	692	461	46	92	230	461	5.0	10.0	24.9	50.0
**3.51**	430	1003	953	903	753	502	50	100	250	501	5.0	10.0	24.9	50.0
**3.62**	456	1076	1023	969	807	568	53	107	269	568	4.9	9.9	25.0	50.0
**3.74**	486	1205	1145	1085	904	603	68	120	301	602	5.6	10.0	25.0	50.0
**3.85**	516	1316	1256	1190	992	661	66	132	330	661	5.0	10.0	25.0	50.0
**3.96**	534	1445	1373	1301	1084	723	72	144	361	722	5.0	10.0	25.0	50.0
